# High throughput accurate method for estimating in vitro dry matter digestibility of maize silage

**DOI:** 10.1186/s13007-021-00788-5

**Published:** 2021-08-11

**Authors:** P.-L. Lopez-Marnet, S. Guillaume, M.-P. Jacquemot, M. Reymond, V. Méchin

**Affiliations:** 1 Institut Jean-Pierre Bourgin, INRAE, AgroParisTech, Université Paris-Saclay, Bat 2 - Route de St Cyr, 78000 Versailles, France; 2 Ecole Doctorale numéro 581: ABIES, AgroParisTech, Université Paris-Saclay, 19 av du Maine, 75732 Paris Cedex 15, France

**Keywords:** In vitro digestibility, Maize silage, Gelatinization, Protocol

## Abstract

**Background:**

Since the introduction of studies on maize silage digestibility at the end of the nineteenth century, protocols to estimate dry matter digestibility have not stopped evolving. Since the early 1980s, the protocol developed by Aufrère became a benchmark in many laboratories to estimate in vitro dry matter digestibility. In order to increase its throughput, to facilitate its execution and to decipher the impact of the different parameters of the protocol we decided to test the combination of 7 parameters in 21 different protocols.

**Results:**

We thus tested the impact of (1) the presence or absence of pepsin in HCl solution, (2) the temperature of incubation during enzymatic hydrolysis, (3) the presence or absence of a gelatinization step, (4) washing/rinsing versus neutralization step, (5) the presence or absence of α-amyloglucosidase in enzymatic solution, (6) the duration of cellulase incubation, and (7) the concentration of the cellulase solution. The major result of our work highlighted that it was essential to carry out a gelatinization step to correctly estimate the in vitro dry matter digestibility of maize silage.

**Conclusions:**

The proposed protocol in this paper is innovative, reliable, highthroughput and easy to implement in many laboratories to accurately quantity in vitro dry matter digestibility.

## Background

First introduction of study on maize silage digestibility appeared in France with the work of Auguste Goffart “Manuel de la culture et de ensilage des maïs” [1] for cow winter nutrition and milk production. During the middle of the twentieth century, the concept of digestibility was first introduced by comparing in vivo silage digestibility and in vitro dry matter digestibility (IVDMD) using rumen fluid [2, 3] (Table 1). Tilley and Terry [2] were the first to propose IVDMD quantification following a two step protocol. The first step consists in the action of rumen fluid directly added on the dry matter (DM) and the second step consists in the action of acidic solution and enzymes [2] (Table 1). However, the use of rumen fluid in protocols caused supply difficulties and its utilization was restricted to few teams [4]. To encompass the use of rumen fluid, IVDMD protocols using fungal enzymes appeared in North America during the 70’s (Table 1). Indeed, in 1975, Jones and Hayward [5] proposed a two step digestibility protocol with fungal enzymes (Table 1). The first step of this protocol consists in an enzymatic pretreatment with pepsin (2 g L^−1^) in acidic solution (HCl 0.1 N) during 24 h at 40 °C and the second step in an acidic digestion with Cellulase (*Trichoderma viride*) during 48 h at 40 °C (Table 1). To established this protocol Jones and Hayward [5] studied the impact of different parameters such as the influence of different pretreatments, the order of the two step, and the impact of different cellulase origins. Their results on grasses demonstrated that a protocol with two step [step 1: Pepsin; step 2: cellulase solution (*T. viride*)] was more accurate (r = 0.96) to account for in vivo digestibility values and variation than a protocol without pepsin pretreatment step (r = 0.91). Moreover, they also showed that applying pepsin solution before cellulase cocktail reflected better in vivo digestibility rather than after cellulase cocktail [5].

**Table 1:** Reported correlations between in vivo and in vitro digestibilities since 1955. Adapted from Aufrere [6] and Marten and Barnes [4]

References	Authors (year)	Forage types	Animals feed	In vitro protocol step 1	In vitro protocol step 2	Correlation coefficient (R)	Prediction equation established
[28]	Thurman and Wehunt (1955)	SorghumCorn silage	Rabbits	–	Solution 1% HCl 15 lb pressure 1 h	0.66	–
[29]	Clark (1958)	Thirteen grass species	Sheep	–	Rumen jus	0.87	–
[2]	Tilley and Terry (1963)	CloverLucerne	Sheep	Rumen jus	HCl 0.1 N2 g L^−1^ pepsin		y = 0.99x − 1.01
[30]	Donefer et al. (1963)	Green grass silage	Sheep	–	Cellulase + pepsin 24 h 40 °C	0.93	–
[14]	Alexander and McGowan (1966)	GrassesLegumesHays	Sheep	Rumen liquor/buffer mixture	HCl–pepsin	0.96	y = 0.97x + 5.05
[31]	Deinum and Soest (1969)	Hays grassesGrass silages	–	–	Rumen jus	0.89	y = 1.28x − 44.8
[32]	Jones and Hayward (1973)	Temperate grasses	Sheep	–	Cellulase *Trichoderma viride* 6.25 g L^−1^ 48 h 40 °C	0.92	y = 0.72x + 32.95
[33]	Hartley et al. (1974)	GrassesLegumes	Sheep	NDF [3]	Cellulase basidiomycete 16 h	0.96	–
				Rumen jus [2]	37 °CHCl 0.1 N2 g L^−1^ pepsin [2]	0.96	–
[5]	Jones and Hayward (1975)	Temperate grass	Sheep	HCl 0.1 N–2 g L^−1^ pepsin 24 h 40 °C	Cellulase “BDH” 48 h 40 °C (*Trichoderma viride*)	0.96	y = 0.61x + 30.4
[34]	McQueen and Van Soest (1975)	Temperate grassLegumes	Sheep and cattle	45 mg Onozuka cellulase in 6.5 m of 1 M acetate. pH 4.5 40 °C 72 h	70 mg pepsin in 1 mL of 1 M acetate. pH 4.5 40 °C 24 h	0.80	–
				Rumen jus [2]	HCl 0.1 N–2 g L^−1^ pepsin [2]	0.95	–
[35]	Adegbola and Paladines (1977)	Tropical grassesLegumes	Sheep	HCl 0.05 NPepsin 48 h 40 °C	Cellulase *Trichoderma* (Worthinton Biochemical Corporation) 48 h 40 °C	0.98	–
[36]	Clark and Beard (1977)	Green SilageHaysStraw	Sheep or cattle	HCl 0.1 NPepsin 24 h 40 °C	Cellulase (*Aspergillus niger*) 48 h 40 °C	0.80	y = 39.1 + 0.76x
[37]	Dowman and Collins (1977)	Grass silage	Sheep	HCl 0.1 NPepsin 24 h 40 °C	Cellulase “BDH” 48 h 40 °C	0.89	y = 0.58x + 31.57
[38]	Goto and Minson (1977)	Tropical and temperate grasses	Sheep	HCl 0.1 NPepsin 48 h 40 °C	Cellulase “Onozuka SS—P” 48 h 40 °C	0.94	y = 0.69x + 20.3
[39]	Roughan and Holland (1977)	Temperate grasses and legumes	–	NDF solution	Cellulase *Trichoderma viride* 5 h and 18 h 50 °C	0.98	y = 0.98x − 10.12
[2]	Tilley and Terry (1963)	Temperate grasses	Sheep	HCl 0.1 N–2 g L^−1^ pepsin 24 h 40 °C	Cellulase *Trichoclerma viride* (“BDH”. phosphate citrate buffer. pH 4.6) 48 h 40 °C	0.96	y = 0.56x + 34.7
[40, 41]	Kellner and Kirchgessner (1977) Kirchgessner and Kellner (1978)	Green silageHaysLegumes	–	HCl 2 N 30 min 100 °C	Cellulase (0.1 M Na-acetate buffer. pH 4.5) 24 h 39 °CHCl 0.1 N—pepsin 2 mg L^−1^ 48 h	0.91	y = 1.15x − 12.02
[42]	McLeod and Minson (1978)	Tropical and temperate grasses	–	HCl 0.1 NPepsin 48 h 39 °C	Cellulase “Onozuka SS—P 1500” 48 h 39 °C	0.94	y = 0.70x + 18.2
[43]	Adamson and Terry (1980)	Hays	Sheep	HCl 0.1 N2 g L^−1^ pepsin 24 h 40 °C	Cellulase *Trichoclerma viride* (“BDH”. phosphate citrate buffer. pH 4.6) 48 h 40 °C	0.91	y = 0.68x + 24.18
[6]	Aufrere (1982)	Green silageHaysLegumes	Sheep	HCl 0.1 N2 g L^−1^ pepsin 24 h 40 °C	Cellulase *Trichoderma viride* “Onozuka R 10” (sodium acetate de 0.05 M pH 4.6)24 h at 40 °C	0.91	y = 0.793x + 0.125 + Δ_Forage_
[7]	Aufrere (1983)	–	–	HCl 0.1 N2 g L^−1^ pepsin 24 h 40 °C30 min 80 °C	Cellulase *Trichoderma viride* “Onozuka R 10” (sodium acetate de 0.05 M pH 4.6)24 h at 40 °C	0.79	–
[8]	Limagrain (1985)	–	–	HCl 0.1 N2 g L^−1^ pepsin 24 h 40 °C30 min 80 °C	Cellulase “Onozuka R 10” and amyloglucosidase 24 h 40 °C	–	–
[16]	Lila et al. (1986)	Maize silage	Sheep	α-Amylase (A-1278 SIGMA) 39 °C 24 hHCl 1 N20 g L^−1^ pepsin 39 °C 24 h	Cellulase (Celluclast 200 L type N de Novo. sodium acetate pH 4.8) 50 °C 58 h	0.25	–
[44]	Aufrere and Michalet-Doreau (1988)	Dry forages, dry roughages, energy feeds, protein supplements	Sheep	HCl 0.1 N2 g L^−1^ pepsin 24 h 40 °C30 min 80 °C	Cellulase *Trichoderma viride* “Onozuka R 10” (sodium acetate de 0.05 M pH 4.6)24 h at 40 °C	0.95	y = 0.699x + 22.6
[45]	De Boever et al. (1988)	Maize silage	Sheep and cattle	HCl 0.1 N—2 g L^−1^ pepsin 24 h 40 °C and 45 min 80 °C	Cellulase 24 h 40 °C	0.71	y = 0.610x + 28.2
[15]	Barrière et al. (1991)	Maize silage hybrids	Texels castrated sheep	Aufrère [6]Lila et al. [16]Limagrain–EnsitecLibramont–Limagrain (NIRS prediction)		0.540.290.380.37	––––
[8, 9]	Andrieu et al. (1993) Dardenne et al. (1993)	Maize silage	Sheep	Limagrain [8]	Limagrain [8]	0.537	–
				Aufrère [7]	Aufrère [7]	0.613	–
				Lila et al. [16]	Lila et al. [16]	0.450	–
[46]	Ferret et al. (1997)	Maize silage	Sheep	Rumen liquor/buffer mixture	HCl–pepsin [14]	0.62	y = 0.75x + 0.1728
[11]	Aufrere (2007)	Green silageSilageHay	Sheep	HCl 0.1 N2 g L^−1^ pepsin 24 h 40 °C30 min 80 °C	Cellulase *Trichoderma viride* “Onozuka R 10” (sodium acetate de 0.05 M pH 4.6)	0.78	y = 0.630x + 29.7
[17]	Damiran et al. (2008)	Forages	Sheep	28 mL of the McDougall’s solution and 7 mL of ruminal fluid 39 °C 48 h	HCl 0.1 N—pepsin 6.6 g L^−1^ 39 °C 48 h(Galyean 1997)	0.58	y = 0.614x + 0.149
[18]	Kowalski et al. (2014)	Grass silage	Sheep	–	Cellulase *Trichoderma viride* “Onozuka R 10” (sodium acetate de 0.01 M pH 4.6) 39 °C 48 h	0.53	y = 0.527x + 0.356
[12]	Peyrat (2015)	Maize	Sheep and cattle	Aufrère et al. (2007)	Aufrère et al. (2007)	0.736	Y = 37.79x + 0.4936
[13]	Virlouvet et al. (2019)	Maize	–	HCl 0.1 N24 h 40 °C	Cellulase *Trichoderma viride* “Onozuka R 10” (sodium acetate de 0.05 M pH 4.6)72 h 50 °C	–	–

Thereafter, even if the different protocols proposed and used by the scientific community have their own specificities, they all retained two step procedure as demonstrated by the protocols reported in Table 1 (adapted from [4, 6]). Aufrère [6, 7] tested several combinations to adapt the digestibility protocol of Jones and Hayward [5] on temperate grasses. During that experimentation, the impact of the 5 following parameters on IVDMD was studied: the acid concentration during the pretreatment step as well as the duration and temperature of this pretreatment, the cellulase concentration in enzymatic treatment step and the addition of a starch gelatinization step to solubilize the high amount of starch provided by the cob in the case of maize dry matter (Table 1) [6, 8, 9]. The starch gelatinization allowed the breaking down of intermolecular bonds of starch granules and more precisely of amylopectin the more crystalline part of starch.

This has led to the establishment of the “Aufrère protocol” [7] to quantify maize IVDMD at silage stage: this protocol first proceeds to a pretreatment step with a 0.1 N HCl solution with 2% pepsin at 40 °C for 24 h. Afterwards a gelatinization step (80 °C for 30 min) is applied. Finally, after filtration and washing, an enzymatic hydrolysis step is carried out at 40 °C with an Onozuka R10 cellulase solution (1 mg mL^−1^) for 24 h. The obtained IVDMD is expressed as the mass percentage of matter lost during these successive steps. Resulting IVDMD quantification obtained with this protocol accounts in an acceptable way for more than 60% of in vivo digestibility observed variation [10].

Nowadays, the protocol developed by Aufrère is a benchmark in many laboratories to estimate IVDMD [8, 9, 11, 12] of silage maize. In its original version, this protocol allows to estimate digestibility but it remains restrictive: first of all because 500 mg of DM are digested and this requires high amount of solutions and space to manipulate and secondly because the gelatinization step is followed by a tedious rinsing step before the addition of enzymatic solution, reducing the throughput of the protocol.

In our group we developed a few years ago a highthroughput protocol to characterize starch-free maize samples digestibility. This protocol tardily published [13] was an adaptation of the Aufrère protocol developed in 1982 for starch-free. This protocol first proceeds to a pretreatment step of 30 mg of dry matter with a 0.1 N HCl solution at 40 °C for 24 h. Afterwards a neutralization step was performed with NaOH 2 N before an enzymatic hydrolysis step carried out at 50 °C with an Onozuka R10 cellulase solution (8 mg mL^−1^) for 72 h.

**Fig. 1:**
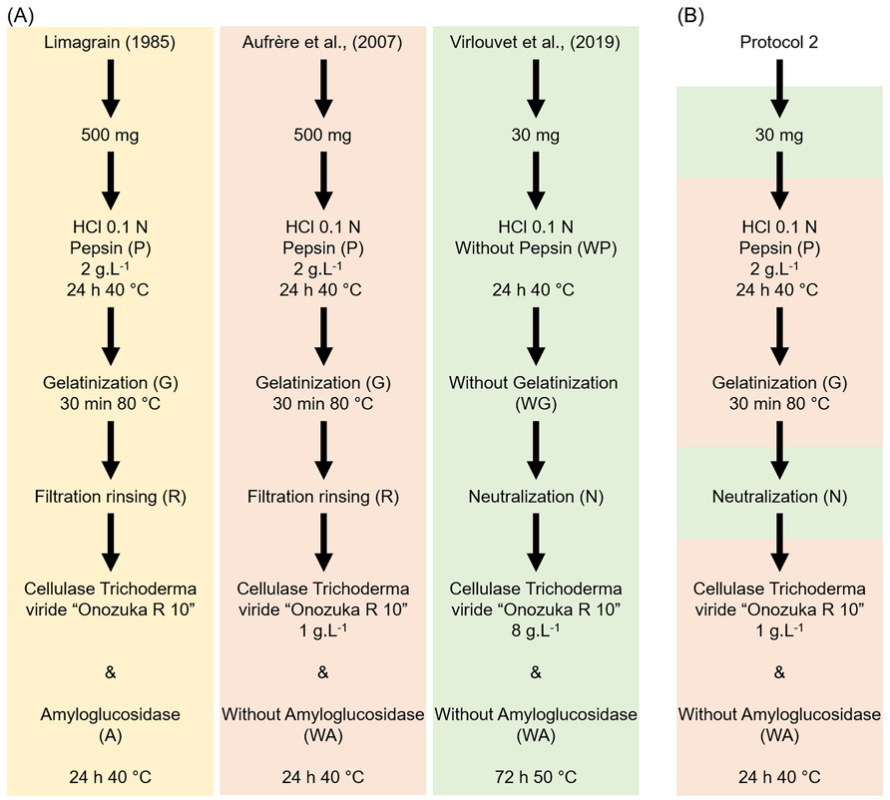
**A** Methodologies for the 3 enzymatic methods selected to test the effect of 7 key parameters (adapted from Andrieu et al. [8]). **B** Protocol 2 adopted following the results presented in this paper as a mixture between the Aufrère et al. (2007) protocol (pink) and Virlouvet et al. [13] protocol (green)

Herein, we developed a protocol dedicated to starch containing maize samples and capable of enabling highthroughput analyzes. With the aim to facilitate protocol execution, we propose to reduce the dry matter sampling to 30 mg and, instead of rinsing, to neutralize the solution after the gelatinization step as proposed in Virlouvet et al. [13]. We also tested the influence of different parameters which differ significantly between Aufrère and Doreau [7] and Virlouvet et al. [13] (Fig. 1a) forthe estimation of IVDMD of maize silage. We also tested the impact of α-amyloglucosidase in enzymatic solution as proposed by Limagrain [8] (Fig. 1a). For that we compared 21 protocols to test the impact of 7 parameters: (1) presence or absence of pepsin in HCl solution, (2) temperature of incubation during enzymatic hydrolysis, (3) presence (G) or absence (WG) of gelatinization step, (4) washing/rinsing (R) or neutralization (N) step, (5) presence or absence of α-amyloglucosidase in enzymatic solution, (6) duration (24 or 72 h) of cellulase incubation, and (7) concentration (1 or 8 mg mL^−1^) of the cellulase solution.

This study leads to the establishment of an accurate, highthroughput and easy to handle protocol to quantify IVDMD of maize samples.

## Results

### Reference values of IVDMD

**Fig. 2:**
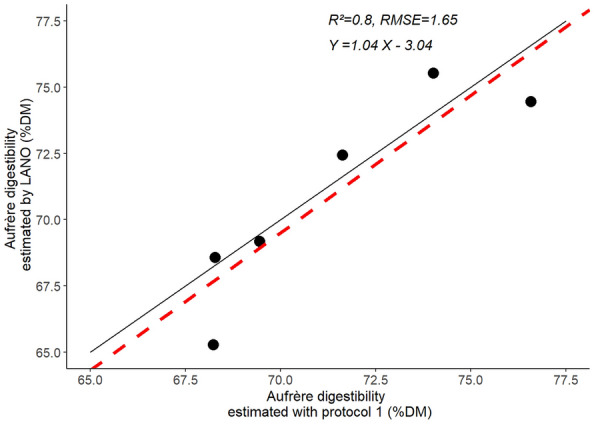
Plot correlation between value of digestibility protocol of two labs. RV is digestibility reference value of LANO labs Aufrere [7] C1 is Aufrere [7] digestibility protocol adapted at IJBP, black line y = x, red dotted lines is the correlation line, R^2^ is the determination coefficient, RMSE is root-mean-square error

IVDMD reference values were determined by LANO laboratory on a selection of 6 maize dry matter samples that cover a large range of variation for dry matter digestibility. These IVDMD reference values varied from 65 to 76%DM (Fig. 2). Protocol 1 (Table 2) is the one which most closely resembles that proposed by Aufrère [6], taken up by the LANO laboratory.

**Table 2:** Protocols analyzed

Protocol number	Pepsin	Gelatinization	Rinsing or neutralization	Cellulase solution (CS)	Amyloglucosidase	Duration of incubation	Temperature of incubation
	2 g L^−1^	80 °C—30 min		[] mg L^−1^	1.5 mL L^−1^	h	°C
1	P	G	R	1	WA	24	40
2	P	G	N	1	WA	24	40
3	WP	WG	N	8	WA	72	40
4	WP	WG	N	8	A	72	50
5	P	WG	N	1	WA	24	50
6	P	WG	N	1	A	24	50
7	P	WG	N	1	A	72	50
8	P	WG	N	8	WA	72	50
9	P	WG	N	8	A	24	50
10	P	WG	N	8	A	72	50
11	P	WG	N	8	A	72	40
12	P	WG	R	1	WA	24	40
13	P	G	N	1	A	24	50
14	P	G	N	8	WA	72	50
15	P	G	N	8	A	24	50
16	P	G	R	1	WA	24	50
17	P	G	R	1	A	24	50
18	P	G	R	8	WA	24	50
19	P	G	R	8	WA	72	50
20	P	G	R	8	A	72	50
21	P	WG	R	8	WA	24	50

The 21 protocols tested as a combination of the 7 retained parameters: (1) Pepsin concentration at 2 (P) or 0 g.L^−1^ (WP), (2) presence of gelatinization (G) or absence (WG), (3) rinsing (R) or neutralization (N) steps, (4) concentration of cellulase solution (8 or 1 mg L^−1^), (5) amyloglucosidase concentration (1.5 (A) or 0 mL L^−1^ (WA)), (6) 24 or 72 h of incubation time and (7) 50 or 40 °C for incubation temperatures

We applied protocol 1 to the 6 selected samples and compared IVDMD obtained values with reference ones. IVDMD values obtained with protocol 1 varied from 67 to 77%DM which faithfully reflects the values obtained by the LANO laboratory. Moreover, between these two protocols, the determination coefficient (R^2^) is 0.8 and the slope is 1.04 (Fig. 2), indicating that IVDMD values obtained with protocol 1 are similar to the ones obtained by the reference protocol [6].

### Gelatinization step is crucial to obtain high similarity of IVDMD with the reference protocol

The impact of 7 parameters was tested by applying on the 6 selected samples the 21 protocols presented in Table 2 which vary for the presence of pepsin, the application of gelatinization, Rinsing or Neutralization, the concentration of cellulase, the presence of amyloglucosidase, the duration of incubation, the temperature of incubation. Determination coefficient, slope and repeatability (cf. “Methods” section) were criteria used to discriminate protocols according to their similarity with the Aufrère protocol [6]. The slope evolved from 0.2 to 1.2 depending protocols. Repeatability is a check of protocol quality and covers a range of 1 to 4% of error except protocol 21 that presents a lower repeatability of with a 6% error.

**Fig. 3:**
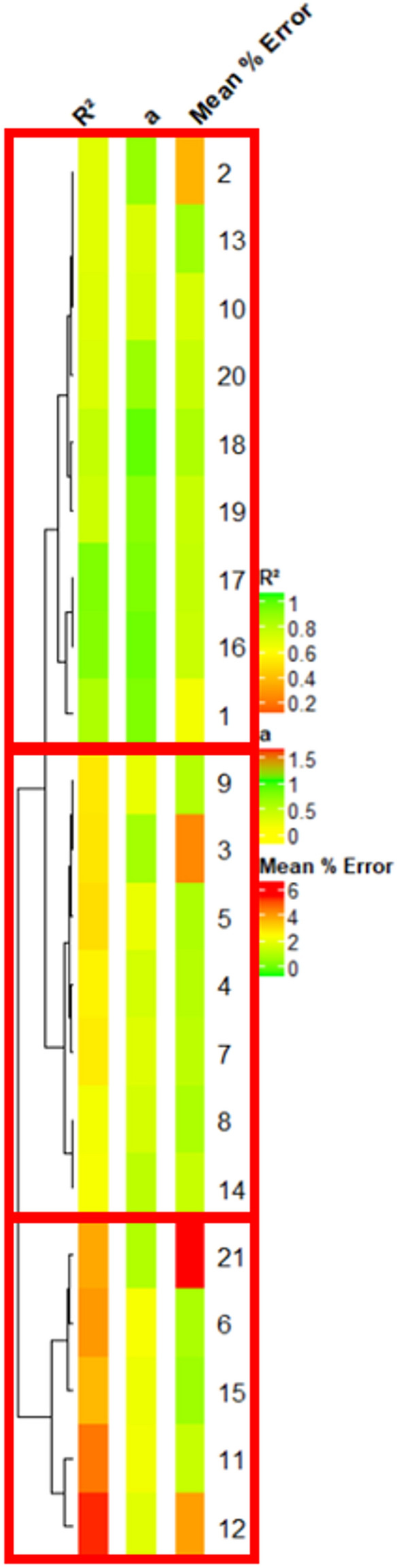
Clustering of the 21 protocols according to their R^2^ and a coefficients. R^2^ is the determination coefficient between Lano reference values and corresponding values obtained by each tested protocols, a is the slope of the correlation between Lano references value and corresponding values obtained by each tested protocols and mean % error is the mean of the values obtained when divided the standard deviation by the mean

**Fig. 4:**
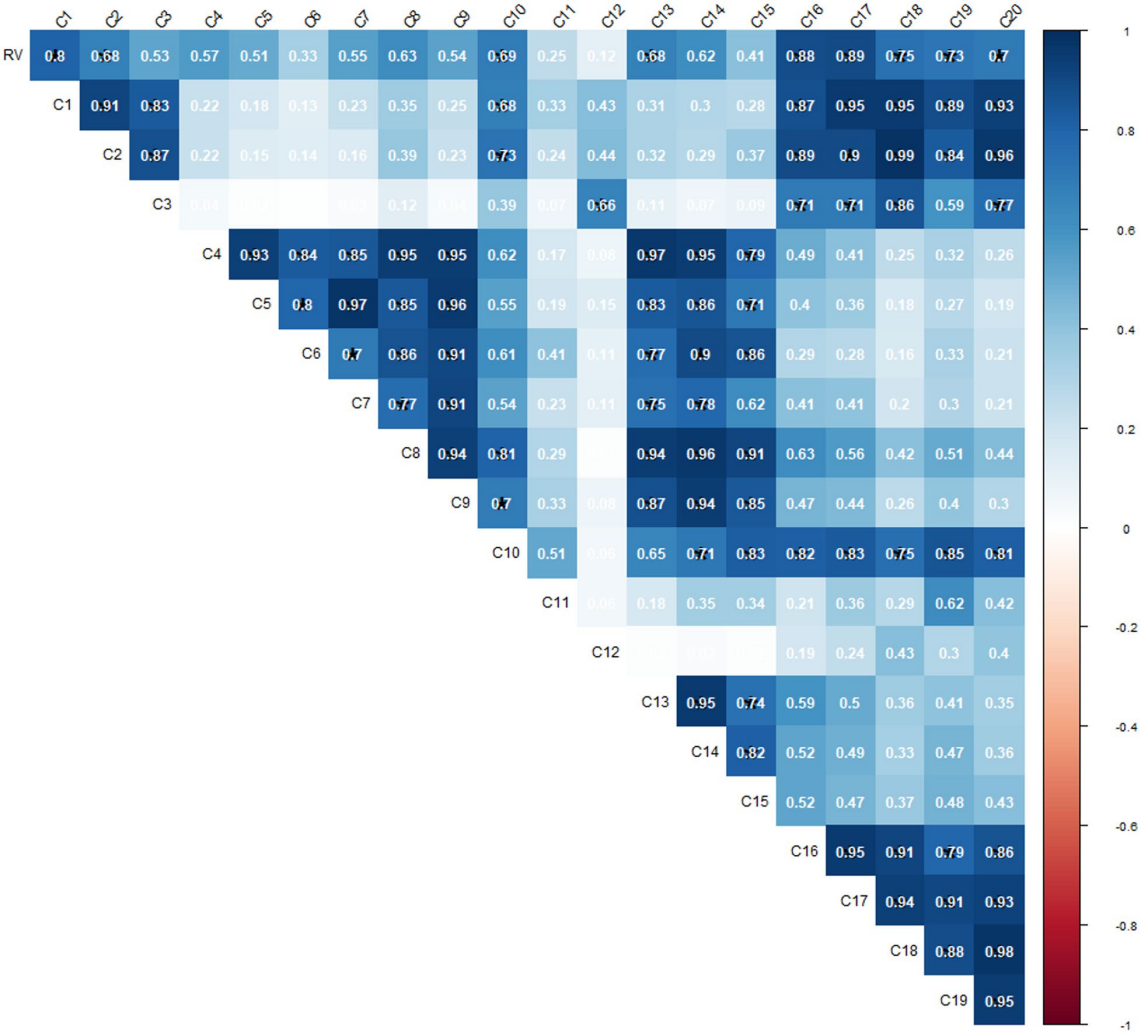
Matrix of correlation between all tested protocols

The dendogram clustered the 21 tested protocols in three distinct groups (Fig. 3). The groups to which Protocol 1 belongs presented highly significant correlations (higher than 0.7) with reference values than the 2 other groups (lower than 0.5) (Figs. 3 and 4). Height of the 9 tested protocols (1, 2, 10, 16, 17, 18, 19 and 20) clustered in this first group are highly correlated together (coefficient correlation R higher than 0.8) (Fig. 4). This group also presented slopes closest to 1. Moreover, protocol 21 belonging to the third group, which showed the highest mean % error (Fig. 3) and was eliminated for further statistical analyses. All other protocols showed good repeatability with less than 5% of mean % error.

**Fig. 5:**
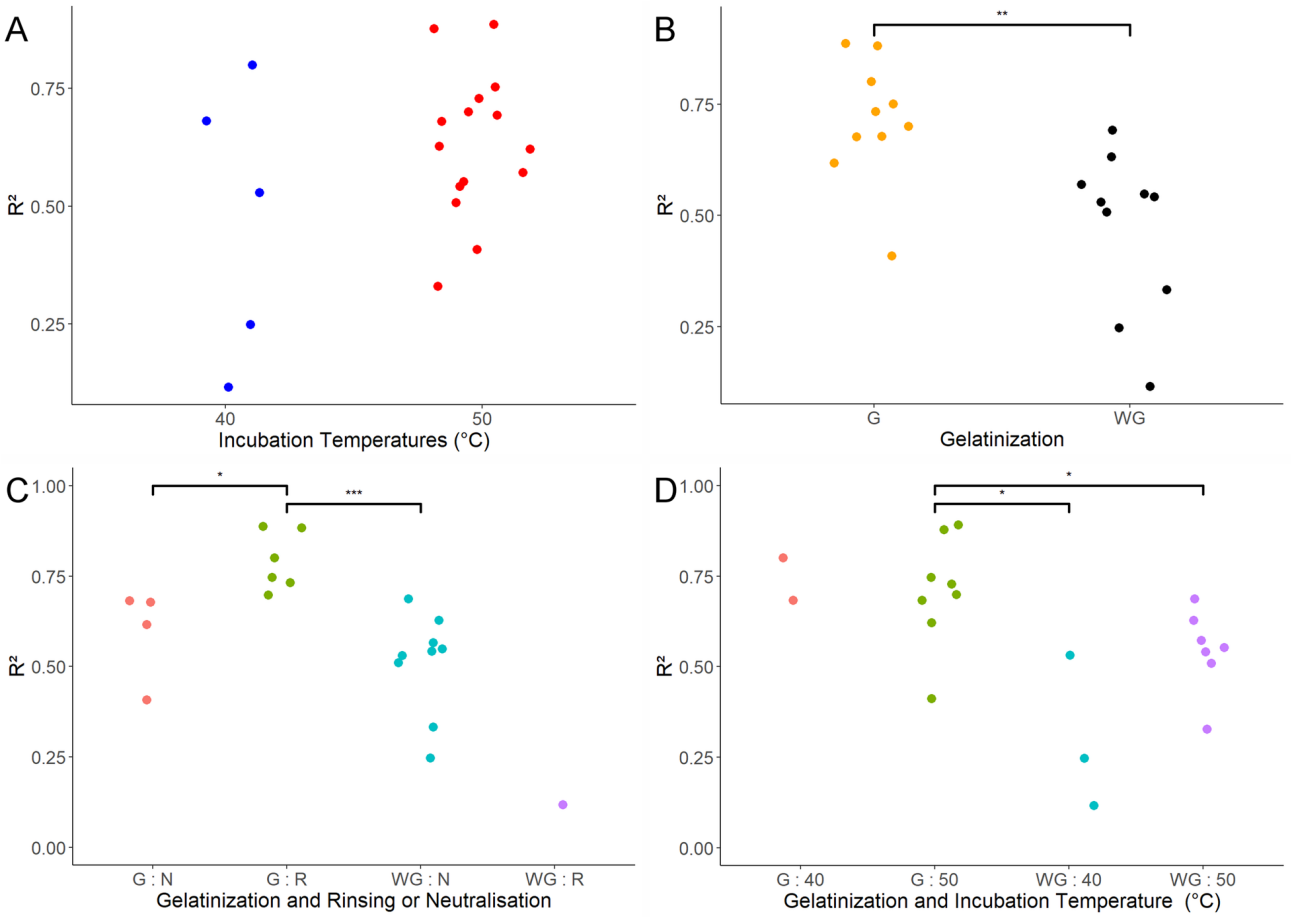
Plot of R^2^ values between reference values and values obtained for each tested protocols sorting according to temperature of incubation (**A**), presence or absence of gelatinization step (**B**), combination of gelatinization and rinsing/neutralization (**C**) and combination of gelatinization and temperature of incubation (**D**). p-value between group of protocols: horizontal lines, ***< 0.001, **< 0.01 and *< 0.05

The regression analysis performed including only main effect parameters yielded in a model with the lowest Akaike Information Criterion (AIC) including the gelatinization step and the temperature of cellulasic hydrolysis incubation (Table 3). In order to clearly depict the impact of these two parameters, R^2^ of protocols were separated either according to their temperature of incubation (40 or 50 °C; Fig. 5A) or according if a gelatinization step was included in the protocol (Fig. 5B). R^2^ of protocols were not significantly different if temperature of incubation was either 40 or 50 °C. On the other hand, R^2^ was significantly increased (pValue = 3.6e10^−3^) when a gelatinization was included in the protocol, suggesting that gelatinization step is clearly a parameter that needs to be included to increase similarity with the Aufrère protocol on maize silage samples.

**Table 3:** Multiple regression analyses with the tested model, selected parameters in the tested model, AIC criteria of the model and p-value of each selected parameters

Model	Step_out	Step_AIC	Anova_p.value	Anova_star
Ymn = µ + Dm + Jr + Emn	Gelatinisation + incubation.temperatures	− 13.28	Gelatinization = 4.48e−04 incubation.temperatures = 1.18e−01	Gelatinization = *** incubation.temperatures = NS
Ymn = µ + Dm + Emn	Gelatinisation	− 12.59	Gelatinisation = 7.33e−04	Gelatinization = ***
Ylmnr = µ + Dm + Jr + Cl + Fn + Lnm + Mmr + Nrl + Elmnr	Gelatinization + incubation.temperatures + pepsin + rinsing.neutralization + gelatinisation:incubation.temperatures + gelatinisation:rinsing. neutralization + incubation.temperatures:pepsin	− 22.94	Gelatinization = 1.43e−06 incubation.temperatures = 3.17e−2 pepsin = 1.41e−1 rinsing.neutralization = 2.94e−1 gelatinisation:incubation. temperatures = 2.83e−3 gelatinisation:rinsing. neutralization = 1.22e−2 incubation.temperatures: pepsin = 2.08e−1	Gelatinization = *** incubation.temperatures:* pepsin = NS rinsing.neutralization = NS gelatinisation:incubation. temperatures = ** gelatinisation:rinsing. neutralization = * incubation.temperatures: pepsin = NS
Ylmnr = µ + Dm + Jr + Lnm + Mmr + Elmnr	Gelatinization + incubation.temperatures + gelatinisation:incubation. temperatures + gelatinisation:rinsing. neutralization	− 22.25	Gelatinization = 3.63e−06 incubation.temperatures = 3.90e−2 gelatinisation:incubation. temperatures = 2.33e−2 gelatinisation:rinsing. neutralization = 5.39e−3	Gelatinization = *** incubation.temperatures = * gelatinisation:incubation. temperatures = * gelatinisation:rinsing. neutralization = **
Ymnr = µ + Dm + Lnm + Mmr + Elmnr	Gelatinization + gelatinisation:incubation. temperatures + gelatinisation:rinsing. neutralization	− 22.25	Gelatinization = 3.63e−06 gelatinisation:incubation. Temperatures = 9.04e−3 gelatinisation:rinsing. neutralization = 5.39e−3	Gelatinization:*** gelatinisation:incubation. temperatures = ** gelatinisation:rinsing. neutralization = **

### Interactions between gelatinization and rinsing/neutralization and between gelatinization and incubation temperature also impacted IVDMD

Multiple regressions were also performed to pinpoint interactions between parameters that impacted the variation of IVDMD protocol compared to the reference protocol. This analysis identified two interactions that significantly impacted the IVDMD estimation (Table 3).

Firstly, the interaction between gelatinization and rinsing/neutralization was involved significantly in the variation of R^2^ of the protocols. Indeed, protocols including a gelatinization step and a rinsing step after gelatinization showed significantly higher R^2^ than protocols without gelatinization and/or with neutralization (Fig. 5C). It is important to note that protocols without gelatinization step and with rinsing step showed similar R^2^ than model with gelatinization and with neutralization step (Fig. 5C).

Secondly, the interaction between gelatinization step and the incubation temperature also significantly impacted the estimation of IVDMD. In order to better understand the impact of this interaction, Fig. 5D showed that the incubation temperature did not impacted the R^2^ of the protocols when a gelatinization step was included. However, without gelatinization, the protocols with incubation temperature set up at 50 °C showed higher R^2^ than protocols with incubation temperature set up at 40 °C (Fig. 5D).

## Discussion

Digestibility protocols have greatly evolved since the middle of the twentieth century. Protocols presented in the literature are very variable. There is no standard in these digestibility estimation protocols and the number of steps, the type or quantity of enzymes or even the incubation times can be very different. In particular, this can make it difficult to compare results from one study to another. Important evolution of these protocols lied in first time by the introduction of two successive steps in the protocols [5, 14] and in second time by the standardization of steps [6, 8, 9, 15]. Some protocols were proposed with a supplemental α-Amylase enzymatic step [16], or McDougall's solution and ruminal fluid (39 °C 48 h) followed by acid pepsin treatment (0.1 N 6.6 g L^−1^ 39 °C 48 h [17]) or only one step of enzymatic hydrolysis by a cellulasic solution [18]. These different protocols gave digestibility values respectively correlated with in vivo* digestibility* to 0.25, 0.58 and 0.53. However, these correlation with in vivo digestibility data were always weaker than correlation obtained with Aufrère 1982 protocol [6]. This was largely described in the literature and coefficient of correlation between in vitro digestibility values and in vivo digestibility evaluation ranged from 0.9 and 0.6. For example this coefficient was of 0.91 in Aufrère [6], 0.54 in Barrière [15], 0.61 in Andrieu and in Dardenne [8, 9], 0.78 in Aufrère [11] and 0.73 Peyrat [12]. The Aufrère protocol is then considered as a robust and reliable protocol but it remains heavy and bulky to implement.

The in vitro digestibility protocol established by Aufrère [6, 10] is the reference one in digestibility studies of maize silage [19]. Moreover in France, combined with protein content, IVDMD values obtained with the Aufrère protocol are used to estimate UFL (Unité Fourrage Laitier) value with model M4 since 1995 [20] and since 2016 with model M4.2 slightly adapted from M4 model [21]. UFL value is essential for the registration process of maize silage varieties in French maize silage catalog [22]. However, the Aufrère protocol does not allow a highthroughput characterization of dry matter samples. Some attempts to adapt the Aufrère protocol to achieve a high characterization rate have been made. The use of Daisy technology have been performed [23]. The Daisy II method can be used on feeds to increase labor efficiency [24] but from our point of view, it still presents major drawbacks. Indeed, this technology always necessitates high quantity of dry matter samples and large volumes of enzymatic solutions. Moreover, the filter bags used in daisy technology can present variations in their porosity depending on the batch used which can cause undigested particle leaks [25]. Furthermore, all the sample is bathed in the same solution and we can always fear interactions between sample and inhibitions on enzymatic activities related to a particular sample. We thus proposed herein, a protocol with smallest dry matter sampling (30 mg) than Aufrère protocol with 500 mg of DM [10].

We demonstrated that few protocols were highly correlated with LANO reference values. With this new format we avoid some problems like variable bag porosity or digestive samples interactions, and it’s broadband, repeatable and comparable to Aufrère protocol. We have clearly underlined the importance of two major parameters in the implementation of these digestibility estimations.

First, we highlighted the need to perform a gelatinization step. This additional step was proposed by Aufrère between 1982 and 1983 [6, 7]. This gelatinization step allowed starch gelatinization i.e. the breaking down of intermolecular bonds of starch granules and more precisely of amylopectin the more crystalline part of starch. Gelatinization step thus allowed a better accessibility of starch molecules for subsequent hydrolysis.

The second important step we revealed is the rinsing step between acidic pretreatment and Onozuka enzymatic hydrolysis step. The combination of these two step (starch gelatinization and rinsing) was the best one to obtain values similar to the reference values. Strictly speaking, we should have retained protocol 16 or 17 as the best one. However, practical aspects allowing highthroughput analyzes also guided our choice. Thus a neutralization step was preferred to rinsing steps even if the results were a little less faithful to the references. This is why protocol 2 was chosen rather than protocol 16 or 17. Protocol 2 combined good statistical performances and presented a high level of practicality for its highthroughput implementation in the laboratory. This combination aimed reducing the arduousness and the time of manipulation while giving very reliable results. Therefore we retained the protocol 2 as the more suitable to easily and accurately measure dry matter digestibility.

## Conclusions

Protocol 2 retained in this study first proceeds to a pretreatment step of 30 mg of dry matter with 0.1 N HCl solution with 2 g L^−1^ pepsin at 40 °C for 24 h. Afterwards a gelatinization step (80 °C for 30 min) is applied. Finally, after a neutralization step, an enzymatic hydrolysis step is carried out at 40 °C with an Onozuka R10 cellulase solution (1 mg mL^−1^) for 24 h (Fig. 1b).

This protocol is innovative on several points compared to the reference protocol of Aufrère and Doreau [7]: the reduction of the dry matter sampling necessary at the start (30 mg versus 500 mg) as well as the neutralization step between the first and the second step, which reduces handling time and improves ease of handling (Fig. 1a, b). This protocol 2 is neither more nor less than the protocol developed by Aufrère and Doreau in 1983 [7] in which the dry matter sampling is reduced to 30 mg, the rinsing replaced by a neutralization step and the glass filtering crucibles replaced by tubes. This allows us to perform experiments with a very large number of samples and reduce volumes of enzymatic solution without hampering the accuracy of the IVDMD quantification.

This protocol is reliable and can be implemented in any laboratory without any specific equipment. It makes it possible to simultaneously characterize a large number of samples, with a small amount of dry matter, with low volumes of solution and without tedious rinsing and filtration steps.

## Methods

### Plant materials

Plants of maize hybrids were cultivated in open field trial in summer 2018. 80 genotypes were grown in two 7 m rows with 0.80 m between rows for 11.2 m^2^ by genotypes and planting density of 90,000 plants/ha. At the silage stage, plants were harvested, dried in an oven (55 °C—72 h) and grind with a hammer mill (1 mm grid). From the 80 harvested samples (one sample per genotype), we selected 6 contrasting samples that cover the range of dry matter digestibility based on results obtained using protocol 1 (Table 2). This set of 6 samples has been used for the analyses carried out in this paper.

### Reference values for IVDMD estimation

Estimation of the IVDMD of the 6 selected samples was performed by the reference laboratory LANO (Laboratoire Agronomique de Normandie http://www.lano.asso.fr/web/index.php) following the initial reference protocol [6, 10]. Briefly, 50 mL of HCl 0.1 N solution with 2% of pepsin was applied on 500 mg of maize dry matter during 24 h at 40 °C. After that, samples were placed 30 min at 80 °C (gelatinization step). Rinsing was performed before adding 50 mL of enzymatic solution of Cellulase Onozuka («Onozuka R-10 from *Trichoderma viride*»; Serva; 10 mg ref 16,419.03) at 1 mg mL^−1^ during 24 h at 40 °C. A final rinsing was then performed before 12 h drying at 103 °C.

### Estimation of IVDM using modified Aufrère protocol

We adapted the Aufrère protocol [10] to our lab conditions and to smaller dry matter sample quantity. This adapted protocol is called Protocol 1 (Table 2) for the rest of the article. Briefly, for this Protocol 1, 30 mg of maize dry matter powder were placed in 5 mL tubes before adding 2 mL of HCl 0.1 N solution with 2% of pepsin during 24 h at 40 °C followed. Then, gelatinization step was carried out by placing the tubes during 30 min at 80 °C in a water bath. The tubes were cooled in ice before being centrifuged at room temperature 10 min at 5000 rpm. The supernatant was discarded. Rinsing was then performed by centrifuging twice with 4 mL of water (5000 rmp—10 min at room temperature) the obtained pellet. After the first rinse centrifugation, the supernatant was discarded and the pellet was rinsed a second time with 4 mL of water. Similarly, the supernatant obtained after the second rinse centrifugation was discarded. 4 mL of Enzymatic solution of Cellulase («Onozuka R-10 from *Trichoderma viride*»; Serva; 10 mg ref 16,419.03) at 1 mg L^−1^ were then added to the pellet, vortexed and agitated during 24 h at 40 °C. The pellet recovered after centrifugation (5000 rmp—10 min at room temperature) was then freezed at − 80 °C and lyophilized 48 h before final weighing.

### Protocols establishment to estimate the impact of different parameters on IVDMD estimation

We tested several protocols to study the influence of 7 parameters on the quantification of dry matter digestibility (Table 2). These 7 tested parameters are as follows:

- presence (2 g L^−1^) or absence of pepsin in HCl solution (0.1 N)
- temperature of incubation (40 or 50 °C) during cellulasic hydrolysis
- presence (G: 30 min at 80 °C) or absence (WG) of gelatinization step
- washing/rinsing (R) or neutralization (N) step. In the case of washing/rinsing alternative, a succession of 3 centrifugation step (5000 rmp, 10 min at room temperature) was performed. The supernatants were discarded. Afterwards, 4 mL of cellulasic solution (concentration according to the protocol) at 4.6 pH were added to the pellet and vortexed. In the case of neutralization alternative, basic solution (90 µL of NaOH solution 2 N) was added in the tube and the tubes were then vortexed.
- presence (1.5 mL L^−1^) or absence of α-amyl glucosidase in cellulasic solution
- duration (24 or 72 h) of cellulasic solution incubation
- concentration (1 or 8 mg mL^−1^) of the cellulasic solution.

All these protocol variants led to the establishment of 21 protocols presented in Table 2. The six selected dry matter samples were digested using these 21 protocols with 3 replicates per protocol.

### Statistical analyses

Means of IVDMD for each sample and for each protocol have been calculated. The repeatability of each protocol was estimated by the mean of the percentage of error (where error = standard deviation/mean) of each sample by protocol.

We analyzed the correlation between each protocol by matrix correlation [26]. Moreover, means of IVDMD values per sample of each protocol were correlated to IVDMD estimation from the LANO laboratory (references values). This allowed us, for each protocol, to obtain a determination coefficient (R^2^) and a slope (“a” coefficient from the equation Y = aX + b, where Y is IVDMD (references values) value and X is IVDMD measured with one of the 21 established protocols (Table 3). R^2^ and slope are reliable indicators to discriminate protocols according to their similarity to the IVDMD reference values from the LANO Laboratory that used the original Aufrère (1982) protocol. The more similar the protocol are and the more the R^2^ values are high and the slope closer to 1. We use the ComplexHeatmap package Rstudio to cluster and visualize the tested protocols according to their R^2^ slope and repeatability values (Fig. 3).

Two multiple regressions analyses were performed using R package “stats” with the “step” functions to.

- analyze main effects of each parameter using the following model:
  $${\text{Ylmnopqr}} =\, \upmu + {\text{Cl}} + {\text{Dm}} + {\text{Fn}} + {\text{Go}} + {\text{Hp}} + {\text{Iq}} + {\text{Jr}} + {\text{Elmnopqr}}$$
- analyze interactions between parameters using the following model:
  $${\text{Ylmnopqr}} =\, \upmu + {\text{Cl}}*{\text{Dm}}*{\text{Fn}}*{\text{Go}}*{\text{Hp}}*{\text{Iq}}*{\text{Jr}} + {\text{Elmnopqr}}$$
  where Ylmnopqr is the R^2^ value of the pepsin concentration “l”, the presence of the gelatinization step “m”, the rinsing or neutralization modality “n”, the cellulasic concentration “o”, the amyloglucosidase concentration “p”, the incubation times “q” and the incubation temperatures “r”. Cl is the main effect of the pepsin concentration “l”; Dm is the main effect of the gelatinization step “m”; Fn is the main effect of the Rinsing or Neutralization “n”; Go is the main effect of the cellulasic concentration “o”; Hp is the main effect of the amyloglucosidase concentration “p”; Iq is the main effect of the incubation times “q”; Jr is the main effect of the incubation temperatures “r”; Elmnopqr is the random residual term.

Wilcoxon test were carried out using R package ggplot2 fonction stat_summary [27] to highlight significant differences between R^2^ values of protocols.

## Data Availability

The datasets used and/or analysed during the current study are available from the corresponding author on reasonable request.

## Abbreviations

IVDMD: In vitro dry matter digestibility
DM: Dry matter
UFL: Unité Fourrage Laitier
